# Quantitative tests reveal that microtubules tune the healthy heart but underlie arrhythmias in pathology

**DOI:** 10.1113/JP277083

**Published:** 2019-01-24

**Authors:** Humberto C. Joca, Andrew K. Coleman, Chris W. Ward, George S. B. Williams

**Affiliations:** 1 Centre for Biomedical Engineering and Technology, University of Maryland School of Medicine, Baltimore, MD, USA; 2 Department of Orthopedics, University of Maryland School of Medicine, Baltimore, MD, USA

## Abstract

Microtubule (MT) mechanotransduction links diastolic stretch to generation of NADPH oxidase 2 (NOX2)-dependent reactive oxygen species (ROS), signals we term X-ROS. While stretch-elicited X-ROS primes intracellular calcium (Ca^2+^) channels for synchronized activation in the healthy heart, the dysregulated excess in this pathway underscores asynchronous Ca^2+^ release and arrhythmia. Here, we expanded our existing computational models of Ca^2+^ signalling in heart to include MT-dependent mechanotransduction through X-ROS. Informed by new focused experimental tests to properly constrain our model, we quantify the role of X-ROS on excitation-contraction coupling in healthy and pathological conditions. This approach allowed for a mechanistic investigation that revealed new insights into X-ROS signalling in disease including changes in MT network density and post-translational modifications (PTMs), elevated NOX2 expression, altered Ca^2+^ release dynamics (i.e. Ca^2+^ sparks and Ca^2+^ waves), how NOX2 is activated by and responds to stretch, and finally the degree to which normalizing X-ROS can prevent Ca^2+^-dependent arrhythmias.

## Introduction

Cardiac excitation-contraction coupling (ECC) is governed by voltage-dependent calcium (Ca^2+^) influx that rapidly activates Ca^2+^ sparks, the elementary Ca^2+^ release events arising from clusters of type 2 ryanodine receptor (RyR2) Ca^2+^ channels in the sarcoplasmic reticulum (SR) membrane (Cheng *et al.* 1993; Cheng & Lederer, 2008). Informed by experimental measures, computational models of Ca^2+^ sparks and cardiac SR Ca^2+^ release have granted further insights into ECC by generating predictions that can inform future experimentation, and provided quantitative characterizations of events that are currently difficult (or impossible) to investigate experimentally (Smith *et al.* 1998; Sobie *et al.* 2002, 2006, 2017; Ramay *et al.* 2011; Williams *et al.* 2011; Wagner *et al.* 2012; Greiser *et al.* 2014; Walker *et al.* 2014; Brandenburg *et al.* 2016; Wescott *et al.* 2016).

**Figure 1.**
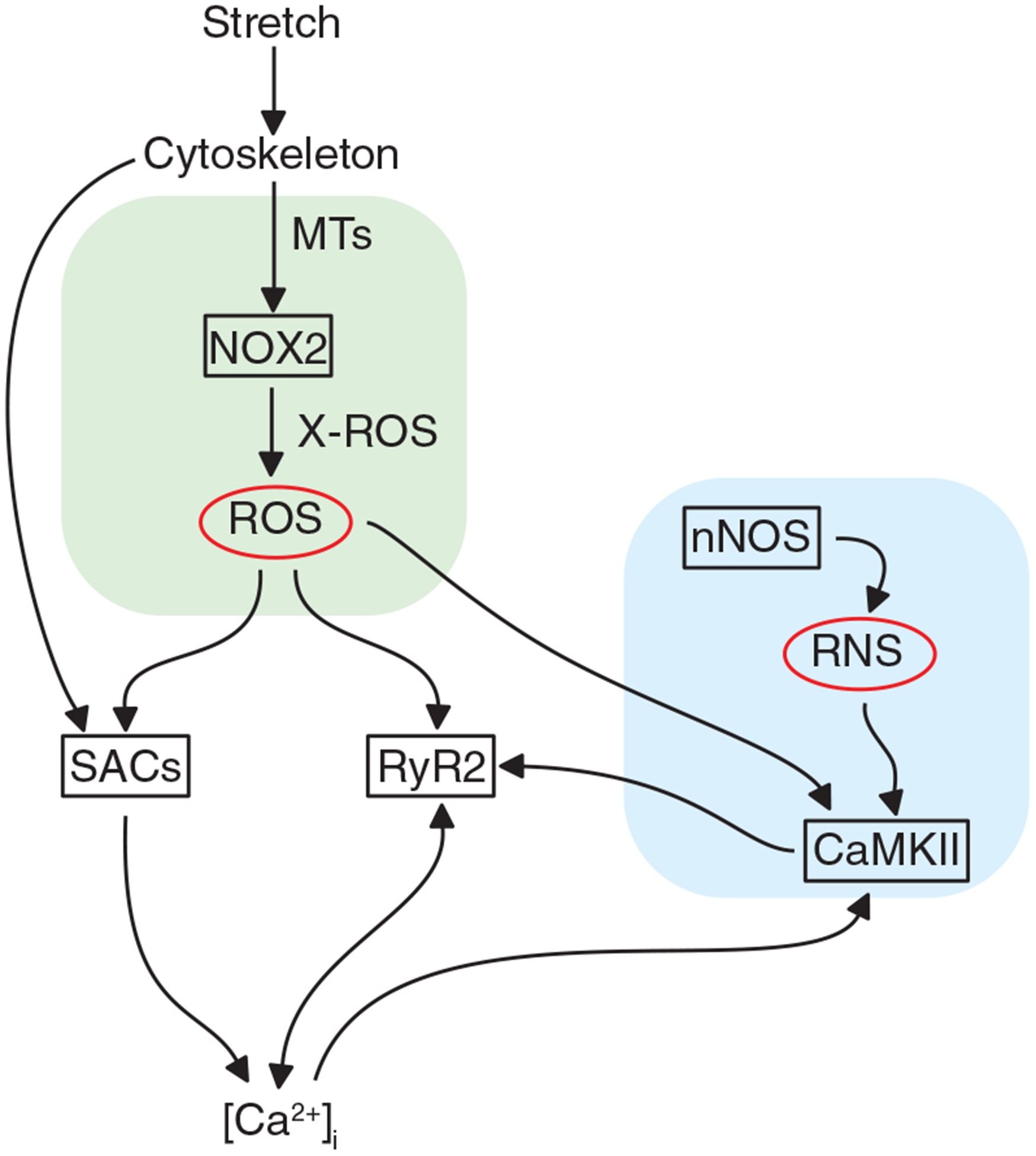
Simplified diagram of stretch-dependent Ca^2+^ signalling in heart [Ca^2+^]_i_, cytosolic [Ca^2+^]; CaMKII, Ca^2+^/calmodulin-dependent protein kinase II; MT, microtubule; nNOS, neuronal nitric oxide synthase; NOX2, NADPH oxidase 2; RNS, reactive nitrogen species; ROS, reactive oxygen species; RyR2, ryanodine receptor, type 2 SAC, stretch-activated channel.

Recent evidence implicates the mechanics of contraction as a regulator of cardiac ECC through mechano-chemo-transduction (MCT) signalling (see Fig. 1; Petroff *et al.* 2001; Niggli *et al.* 2013; Jian *et al.* 2014). While reactive nitrogen species (RNS) have been implicated as a MCT-generated signal regulating RyR2 and sarcolemmal stretch activated channels (SACs; i.e. TRPs), an early report showed that a brief diastolic stretch elicited Ca^2+^ sparks independently of regulation by RNS or SACs (Iribe *et al.* 2009). Guided by our initial evidence that MCT through microtubules (MTs) drove Ca^2+^ spark activation during diastolic stretch (Iribe *et al.* 2009) and earlier discoveries implicating MTs in regulating ECC in heart (Tsutsui *et al.* 1993; Tagawa *et al.* 1998), we focused our attention on the mechanisms by which MTs regulate Ca^2+^ spark activation during stretch, seeking to add quantitative details to the pathway through a comprehensive computational model.

Taken together, our evidence shows that MCT through MTs activates NADPH oxidase 2 (NOX2)-derived reactive oxygen species (ROS), signals we termed X-ROS (Prosser *et al.* 2011). We showed MT-dependent MCT through X-ROS is encoded in the frequency (i.e. heart rate) and amplitude (i.e. preload) of stretch and that MTs stabilized by detyrosination, an enzymatic post-translational modification (PTM) of the *α*-tubulin, governs this effect (Kerr *et al.* 2015). Importantly, our work in dystrophic cardiomyopathy links the disease-dependent increase in MT proliferation, level of detyrosination and NOX2 expression to the excess Ca^2+^ spark activation linked to arrhythmia and disease progression. Consistent with the proximate role of MTs in the activation of X-ROS, targeting the disease-altered MT network (Kerr *et al.* 2015) is solely sufficient to abrogate the deleterious excess in stretch-activated Ca^2+^ spark activity *in vitro* as well as workload-elicited Ca^2+^-dependent arrhythmia *in vivo*.

Here, we have extended these discoveries and show that X-ROS sensitizes stretch activated channels (SACs), a result consistent with previous reports implicating ROS as regulators of SACs in healthy and diseased heart (Jung *et al.* 2008; Kerr *et al.* 2015). Informed by published work and the new results presented here, we have expanded upon and improved our existing computational models of cardiac ECC. These efforts have yielded a computational model integrating MT-dependent MCT and cardiomyocyte ECC through X-ROS. This new computational model is a powerful platform that lays the foundation for future investigations into MCT’s influence on cardiac ECC under both physiological and pathological conditions.

## Methods

### Ethical approval

Animal care and procedures were approved and performed in accordance with the standards set forth by the University of Maryland, Baltimore, Institutional Animal Care and Use Committee (Approval no. 0617015) and the *Guide for the Care and Use of Laboratory Animals* published by the US National Institutes of Health (NIH Publication, 8th edn, 2011). The study conformed to the principles and regulations of *The Journal of Physiology* (Grundy, 2015). WT (e.g. C57/B6) and MDX (D2.B10-Dmdmdx/J)) mice were purchased from The Jackson Laboratory (Bar Harbor, ME, USA). Of note, this dystrophin-deficient murine model on the DBA2 background presents with a more severe phenotype than seen in the MDX mice (C57BL/10ScSn-Dmdmdx/J) we profiled previously. All animals had access to food and water *ad libitum* and were exposed to a 12 h light-dark cycle. Mice were deeply anaesthetized with isoflurane anaesthesia (3%), delivered by a veterinary grade vaporizer. Euthanasia was by rapid exsanguination secondary to the removal of the heart.

### Cardiomyocyte isolation

Adult, male mice (8–16 weeks) were anaesthetized by isoflurane vapour, followed by the excision of the heart and enzymatic isolation of ventricular cardiomyocytes (VCMs) as previously described (Shioya, 2007). Cardiomyocytes were stored in a normal Tyrode solution containing (in mM): NaCl 140, KCl 5, CaCl_2_ 1.8, MgCl_2_ 0.5, HEPES 5, glucose 5, NaH_2_PO_4_ 0.33 (pH 7.4). Experiments were performed at room temperature (25°C).

### Immunostaining and western blot

Microtubule and sarcomere structure were profiled in cells fixed in paraformaldehyde (4%) for 15 min, followed by 10 min permeabilization with Triton X-100 (0.1%). SuperBlock™ phosphate-buffered saline (PBS) was used for blocking procedure and antibody dilution (Thermo Fisher Scientific, Waltham, MA, USA). The cells were incubated overnight (4°C) with mouse *α*-tubulin antibody (Millipore Sigma, St Louis, MO, USA) and phalloidin conjugated with Alexa Fluor 633 (Thermo Fisher Scientific), followed by a 2 hour incubation in secondary anti-mouse antibody conjugated with Alexa Fluor 488 (Thermo Fisher Scientific). VCMs were imaged using a Nikon A1R inverted confocal microscope with a ×60/1.4 NA oil-immersion objective (Nikon, Melville, NY, USA).

The free and polymerized tubulin fractions were isolated from snap frozen hearts as in Belanto *et al.* (2016), with western blots for *α*-tubulin and its detyrosinated form (i.e., glu-tubulin, the enzymatic removal of the C-terminal tyrosine yielding a terminal glutamate), as described (Kerr *et al.* 2015).

### Axial stretch of VCMs

All experiments were performed in custom-fabricated cell chambers mounted on an inverted microscope. The diastolic stretch was performed as previously described (Prosser *et al.* 2013b; Kerr *et al.* 2015). Briefly, glass cell holders (Ionoptix, Milton, MA, USA) were coated with a biological adhesive, MyoTak™ (Ionoptix). One glass cell holder was connected to a length controller (Nano-OP65, Mad City Labs, Madison, WI, USA) to control the stretch paradigms. Myocytes were attached at both ends by gently pressing down with the MyoTak-coated holder and then lifting the cell from the chamber bottom. Axial stretch was applied by movement of the length controller in response to variable voltage waveform controlled by software (Aurora Scientific, Ontario, Canada). Also, the sarcomere length was monitored with a high-speed video camera (Aurora Scientific). Average sarcomere length prior to stretch was 1.8 *μ*m. To assure proper attachment and stretch amplitude, the VCMs were subjected to stretch-relaxation trials before the experiments. Experimentation only proceeded with well-attached VCMs. Similar sarcomere length changes are achieved and maintained with static and 2 Hz cyclic (sinusoidal waveform) stretch (Prosser *et al.* 2011, 2013b; Kerr *et al.* 2015). Only cells with no signs of damage or disturbed integrity of cell membranes in response to any of the stretch paradigms were included in this study.

### Ca^2+^ spark measurements

Cells were loaded with Fluo-4 by 15 min incubation with 3 *μ*M of Fluo-4-acetoxymethyl (AM) ester (Thermo Fisher Scientific) and 0.01% Pluronic F127 (a poloxamer made by BASF, Florham Park, NJ, USA) followed by an additional 10 min for de-esterification. Cells were scanned using a 488 nm laser in a Nikon A1R inverted confocal microscope with a ×40/1.3 NA oil-immersion objective. The acquisition was performed in confocal line-scan mode at 1.84 ms per line. Automated analysis of line-scan images for Ca^2+^ spark location and properties were performed using ImageJ (National Institutes of Health, Bethesda, MD, USA) with SparkMaster plugin (Picht *et al.* 2007).

### Manganese quench measurements

To determine the relative magnitude of Ca^2+^ influx, we adopted and modified a ‘manganese (Mn) quench’ technique as we previously described (Kerr *et al.* 2015). In this method, the equimolar replacement of Ca^2+^ with Mn^2+^ in normal Tyrode solution results in Mn^2+^ permeation through Ca^2+^-permissive cation channels, which quenches the Fura-2 fluorescence (*E*_x_: 360 nm, *E*_m_: 510 nm). Here myocytes were loaded with 5 *μ*M Fura-2 AM (Thermo Fisher Scientific) and 0.01% Pluronic F127 (see above) for 15 min followed by 10 min for de-esterification. Cells were imaged using an IX70 inverted fluorescence microscope (Olympus, Center Valley, PA, USA) with a ×40/1.1 NA water-immersion objective. The Fura-2 was excited at its isobestic point using a 360 nm light from the DG-4 illumination system (Sutter Instrument, Novato, CA, USA) and the emission was captured with a sCMOS camera (pco.edge 4.2, PCO AG, Kelheim, Germany) each second. Prior to stretch trial, the perfusion with normal Tyrode solution was switched to a Mn-Tyrode solution to establish the basal Mn permeability (30 s). Cells were then stretched for 60 s with a sinusoidal waveform (2 Hz; 10% cell length) as described previously (Prosser *et al.* 2011, 2013b; Kerr *et al.* 2015), followed by a return to resting sarcomere length (SL) (30 s). To evaluate the impact of ROS on the influx pathway, cells were incubated with normal Tyrode solution supplemented with 10 mM of *N*-acetyl l-cysteine (NAC; Millipore Sigma) for 5 min, followed by a second trial of Mn^2+^ quenching and 2 Hz cyclic stretch.

### Statistics

**Figure 2.**
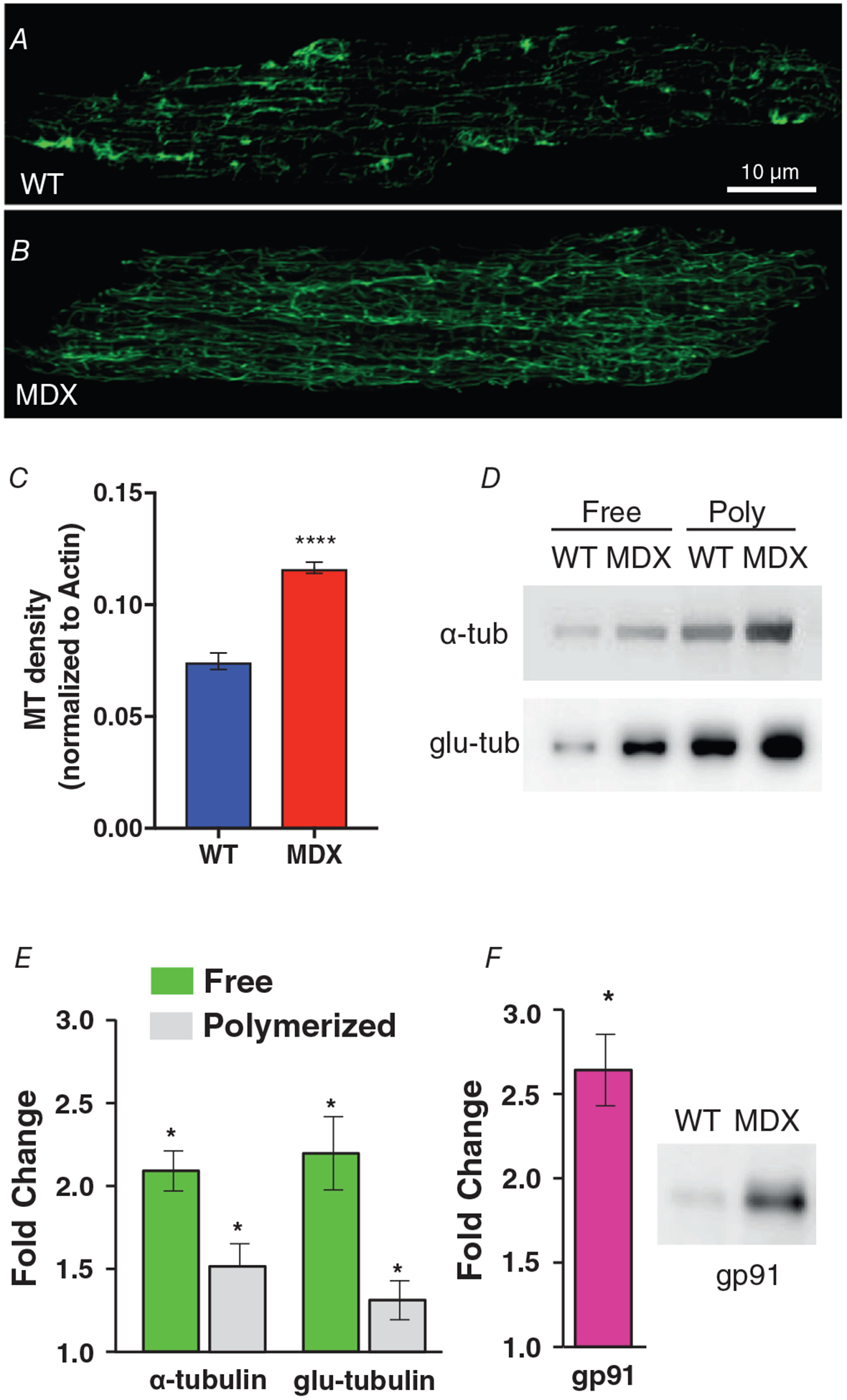
Microtubules in heart *A* and *B*, VCMs from WT (*A*) and MDX (*B*) hearts immunolabelled for *α*-tubulin. *C*, quantification of MT density was obtained by normalizing the skeletonized binary area of *α*-tubulin (*A* and *B*) over the binary area of actin (not shown). *D*, western blots for free and polymerized fraction of a-tubulin and glu-tubulin, respectively. *E*, quantification of western blot in MDX hearts showing the overexpression of tubulin proteins over WT hearts. *F*, western blots and quantification (fold change) for total gp91 expression in WT and MDX. ****P < 0.001, *P < 0.05 over WT group.

**Figure 3.**
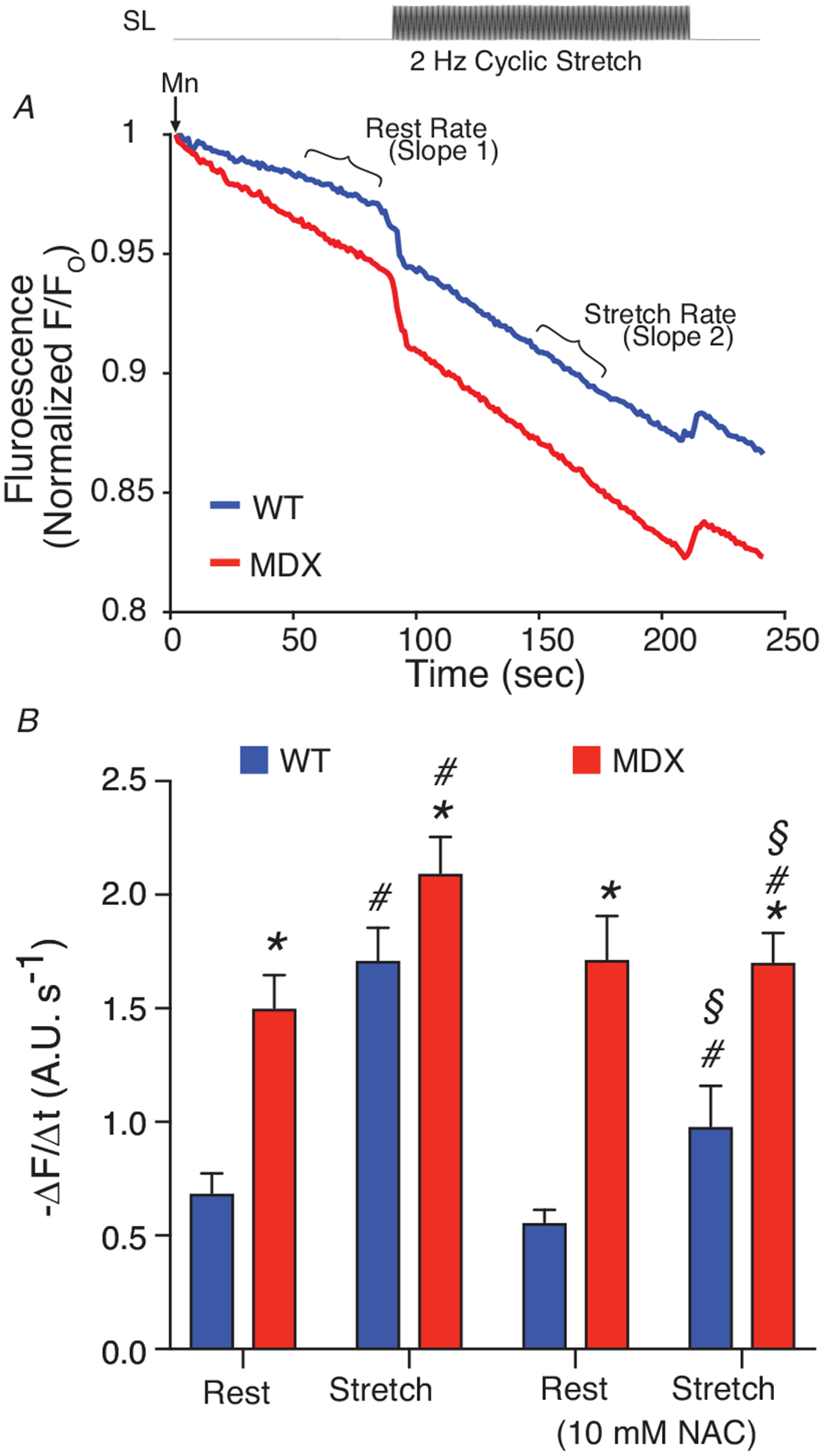
Physiological stretch alters Ca^2+^ entry in heart VCMs are first attached to glass cardiac fingers (see Methods). VCMs are loaded with Fura-2 AM (5 *μ*M, 15 min). *A*, extracellular Ca^2+^ (1.8 mM) is replaced with equimolar Mn^2+^ which enters the cell via sarcolemmal Ca^2+^ channels and quenches Fura-2 florescence (see Slope 1). During a 2 Hz cyclic stretch, elevated Ca^2+^ influx increases the quench rate (see Slope 2). *B*, analysis of Ca^2+^ influx rates. Statistics: MDX compared to WT (*P < 0.05), Stretch compared to Rest (^#^*P* < 0.05), and NAC compared to vehicle (^§^*P* < 0.05).

**Figure 4.**
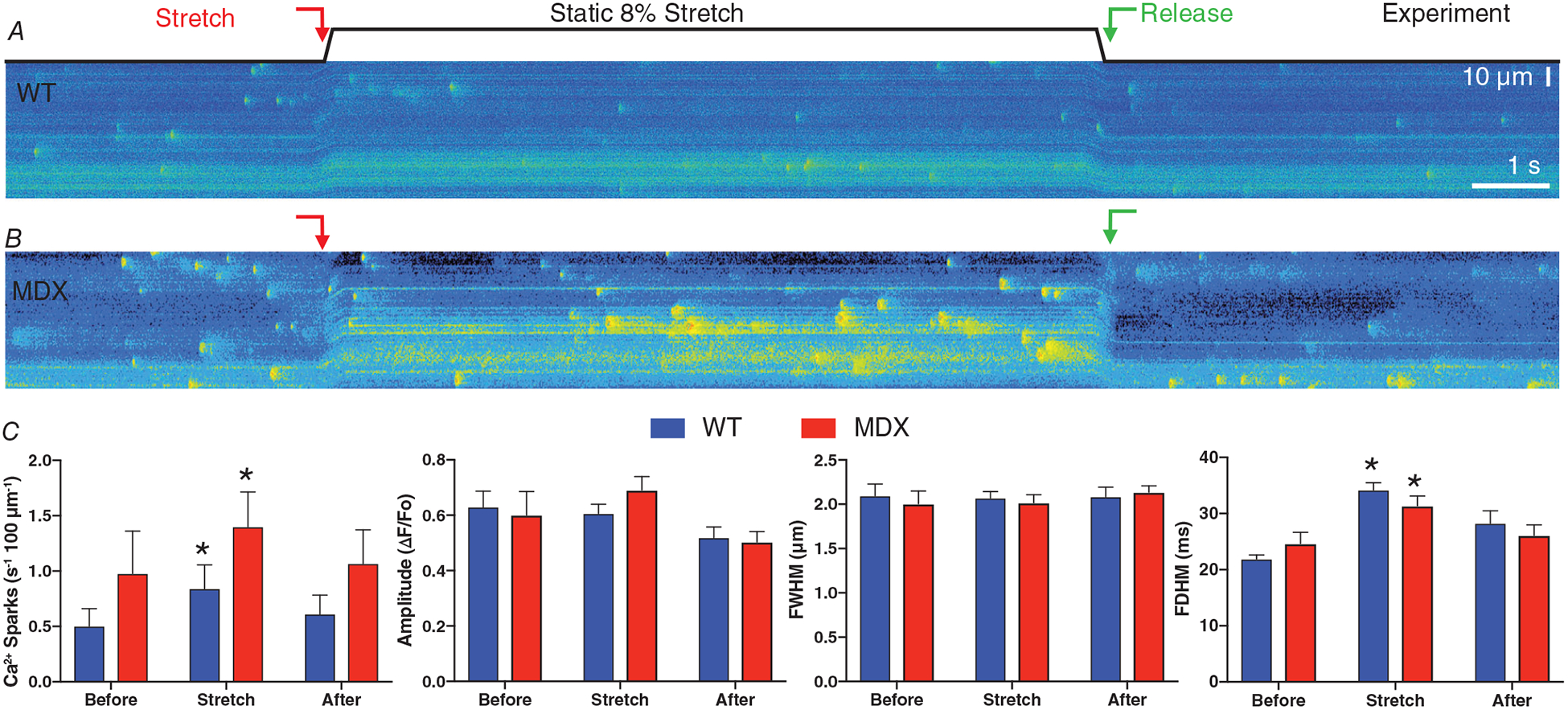
Stretch-dependent changes in Ca^2+^ signalling in heart *A* and *B*, representative, longitudinal confocal linescans for WT (*A*) and MDX (*B*) VCMs loaded with Fluo-4 AM. *C*, Ca^2+^ spark frequency, amplitude, FDHM and FWHM before, during and after a static, 8% stretch. Statistics: Stretch compared to Before (*P < 0.05).

All the data are presented as mean value ± standard error of the means (SEM) unless otherwise noted. Following a test for normality, either Student’s unpaired *t* test (for two groups) or one/two-way ANOVA (for more than two groups) was used for normally distributed data (Figs 2, 3 and 7). Data with a non-normal distribution was analysed with a Kruskal-Wallis one-way ANOVA within each genotype (Ca^2+^ spark full width, half-maximum (FWHM), full duration, half maximum (FDHM) and amplitude, Fig. 4) or with Friedman’s repeated measures ANOVA across all groups (Ca^2+^ spark frequency, Figs 4 and 6) with a corrected Bonferonni *post hoc* test for multiple comparisons (SigmaStat v 4.1 (Systat Software, Inc., San Jose, CA, USA) and SPSS Statistics v 26 (IBM Corp., Armonk, NY, USA)).

### Model formulation

The computational models of Ca^2+^ signalling in heart utilized here are based on published work by our group (Williams *et al.* 2011; Walker *et al.* 2014; Wescott *et al.* 2016). The compartmental Monte Carlo model used here consists of ordinary differential equations (ODEs) representing the time-evolution of various intracellular ion concentrations (i.e. [Ca^2+^]_i_, [Na^+^]_i_, [K^+^]_i_,) and ion current gating variables. These ODEs are then coupled to two discrete-state, continuous-time Markov chains representing the stochastic gating of the LCC and RyR2. See Wescott *et al.* (2016) for more details. For the purposes of this study, the cell’s sarcolemmal membrane was clamped to −80 mV to simulate a quiescent cardiomyocyte. The 3-D, spatial model of Ca^2+^ diffusion utilized consists of four reaction-diffusion style, partial differential equations which govern the diffusion of free cytosolic Ca^2+^, Ca^2+^ bound to Fluo-4, free SR Ca^2+^ and Ca^2+^ bound to Fluo-5N over the simulated region. This spatial region was designed to mimic a transverse confocal linescan of a ventricular cardiomyocyte. Within this spatial domain, Ca^2+^ release units (CRUs) are randomly distributed along the transverse direction with a mean inter-CRU distance of 0.6 *μ*m. Details regarding channel gating were identical between the compartmental and spatial formulations. See Walker *et al.* (2014) and Wescott *et al.* (2016) for more details.

#### Simplified X-ROS formulation

Based on results from our group and others, we have focused modelling MT-dependent MCT via three primary components that activate during sarcomere stretch: (1) mechanical strain within MTs activates NOX2; (2) NOX2 generates X-ROS signals; and (3) X-ROS signals sensitize the activation of RyR2 and SACs (for review see Prosser *et al.* 2013a). We have delimited our model to address these components.

##### Sarcomere length

During stretch, current SL (SL) is governed by:

(1) $${\text{SL=SL}}_{\text{1}}{+A}_{\text{s}}\text{sin}\left(2\pi \omega t-\pi /2\right)$$

where SL_1_ = 1.92 *μ*m, and *A*_s_ = 0.08 *μ*m.

##### NOX2 activity

Here we present a discrete-state, continuous-time Markov chain designed to simulate the activity of NOX2 in heart. This Markov chain is represented by the following three-state transition-state diagram, where D represents the ‘deactivated’ state, A the ‘activated’ state, and R the ‘refractory’ state.
(2)
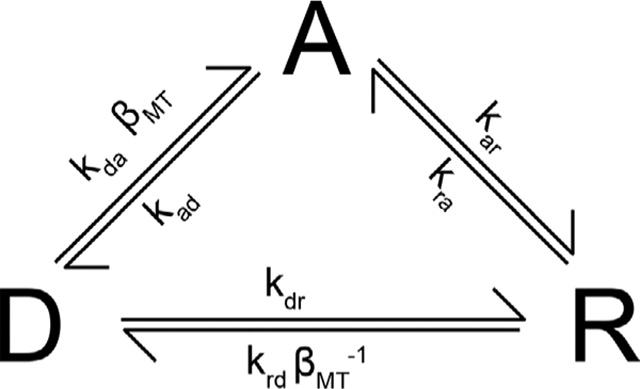

where each *k* (e.g. *k*_da_, *k*_ad_, etc.) is a rate constant with units of s^−1^ (see Table 1) and *β*_MT_ is a unitless ‘MT strain’ factor for NOX2 given by:

(3) $$\beta_{\text{MT}}=H\left[\text{SL}-{\text{SL}}_{\text{R}}\right]{\left(\beta_{\text{m}}\frac{\text{SL}/{\text{SL}}_{\text{R}}-1}{\beta_{1}}\right)}^{\beta_{\eta }}+\beta_{0}$$

where *H* is the heaviside function, SL_R_ is the resting sarcomere length, *β*_m_ is a MT strain factor scalar, *β*_n_ is MT strain cooperativity factor, *β*_1_ is the maximal MT strain factor, and *β*_0_ is the minimal MT strain factor (see Table 1).

##### RyR2 sensitization by ROS

Previously established RyR2 gating rate constants are scaled by an ‘oxidization’ factor (*χ*) which is given by:

(4) $$\chi =1+\left(\rho_{\text{X}}-\rho_{\text{X}}^{o}\right)\pi_{\text{X}}^{A}-\rho_{\text{X}}^{o}$$

where *ρ*_X_ is an ‘X-ROS scalar’ formed by the relative densities of MTs and NOX2 (*ρ*_MT_ and *ρ*_NOX2_, respectively) such that *ρ*_X_ = *ρ*_MT_*ρ*_NOX2_, *ρ*_X_^o^ represents a basal oxidization factor, and *π*_X_^A^ is the probability of NOX2 being activated (in state ‘A’ from above). Each RyR2’s gating is determined by the following two-state, continuous-time Markov chain represented by the following transition-state diagram,

(5) $$\left(\text{Closed}\right)\;C\;\overset{\chi k_{\text{12}}c_{\text{ds}}^{n}\phi }{\underset{k_{\text{21}}/\chi^{\text{2}}}{⇌}}\;O\;\left(\text{Open}\right)$$

where *k*_12_ is the basal RyR2 opening rate in *μ*M^−*η*^ s^−1^, Φ is a luminal regulation function (see Wescott *et al.* 2016), *c*_ds_ is the subspace [Ca^2+^], *η* is the RyR2 Ca^2+^-binding cooperativity constant and *k*_21_ is the intrinsic RyR2 closing rate in s^−1^.

While simple and computationally efficient, this formulation accurately captures both the activation of NOX2 and how NOX2-produced ROS alters RyR2 gating during stretch across both static and cyclical stretch protocols (see Results).

##### Stretch-dependent Ca^2+^ influx in heart

Evidence suggests that sarcolemmal stretch-activated channels (SACs; i.e. TRPs) contribute to the dynamic regulation of Ca^2+^ in cardiomyocytes (Zeng *et al.* 2000; Kondratev *et al.* 2005; Aguettaz *et al.* 2016). A stretch-activated Ca^2+^ influx (SACa) current was added to the existing computational model and was constrained by Ca^2+^ influx measurements shown here (see Fig. 4). This Ca^2+^ current takes the following form:

(6) $$I_{\text{saca}}=\frac{{\left(\chi -1\right)}^{2}}{{\left(\chi -1\right)}^{2}+{\left(K_{\text{d}}^{\chi }\right)}^{2}}g_{\text{saca}}\left(V-E_{Ca}\right)$$

where *K*_d_^X^ is the sensitivity coefficient for activating *I*_saca_, *g*_saca_ is the whole conductance for the current, *V* is the sarcolemmal membrane voltage (in mV), and *E*_Ca_ is the Nernst reversal potential for Ca^2+^.

## Results

Having established the formulation for our computational model, we next performed a focused series of experimental tests to expand our understanding of three main areas of stretch-dependent Ca^2+^ signalling in the heart. First, we quantified the alterations in MT network associated with disease (i.e. MDX), then we characterized the amount of Ca^2+^ that enters a VCM during stretch, and finally how Ca^2+^ spark properties are altered during stretch in both healthy and diseased hearts. The computational model was constrained and simulations were performed to assess the ability of the model to make predictions regarding MT-driven changes in Ca^2+^ signalling.

### MT density, levels of detyrosination and NOX2 expression are increased in disease

We have previously linked both the increase in MT densification and its stabilization by detyrosination as well as the increased NOX2 expression, to the increased X-ROS generation in the dystrophic heart (Kerr *et al.* 2015). Here we have characterized and quantified the changes in MTs and NOX2 and used the results to improve our computational model of X-ROS. VCMs from WT and MDX mice were immunolabelled with antibodies (Abs) against *α*-tubulin (Fig. 2A and B) and phalloidin to visualize actin (not shown). We showed MDX VCMs have an ~50% increase in MT density compared to WT (Fig. 2C), confirmed by western blot quantification of the polymerized MT fraction from intact hearts, which showed a 1.5-fold increase in polymerized MT fraction, confirming this finding. Free tubulin was also increased in the dystrophic hearts, consistent with an increase in tubulin expression further contributing to this effect. Additionally, there was a proportional increase in glu-tubulin in the polymerized MT fraction, which we have shown is essential to MT-dependent MCT (Kerr *et al.* 2015)). Together, these changes are qualitatively similar to those we have previously shown in hearts from aged MDX mice. Western blot quantification of WT *vs*. MDX also revealed an ~2.5-fold increase in gp91phox, the catalytic subunit of NOX2. Again, this increase was similar to our previous work in the aged MDX (Kerr *et al.* 2015).

### Stretch-activated Ca^2+^ influx

**Figure 5.**
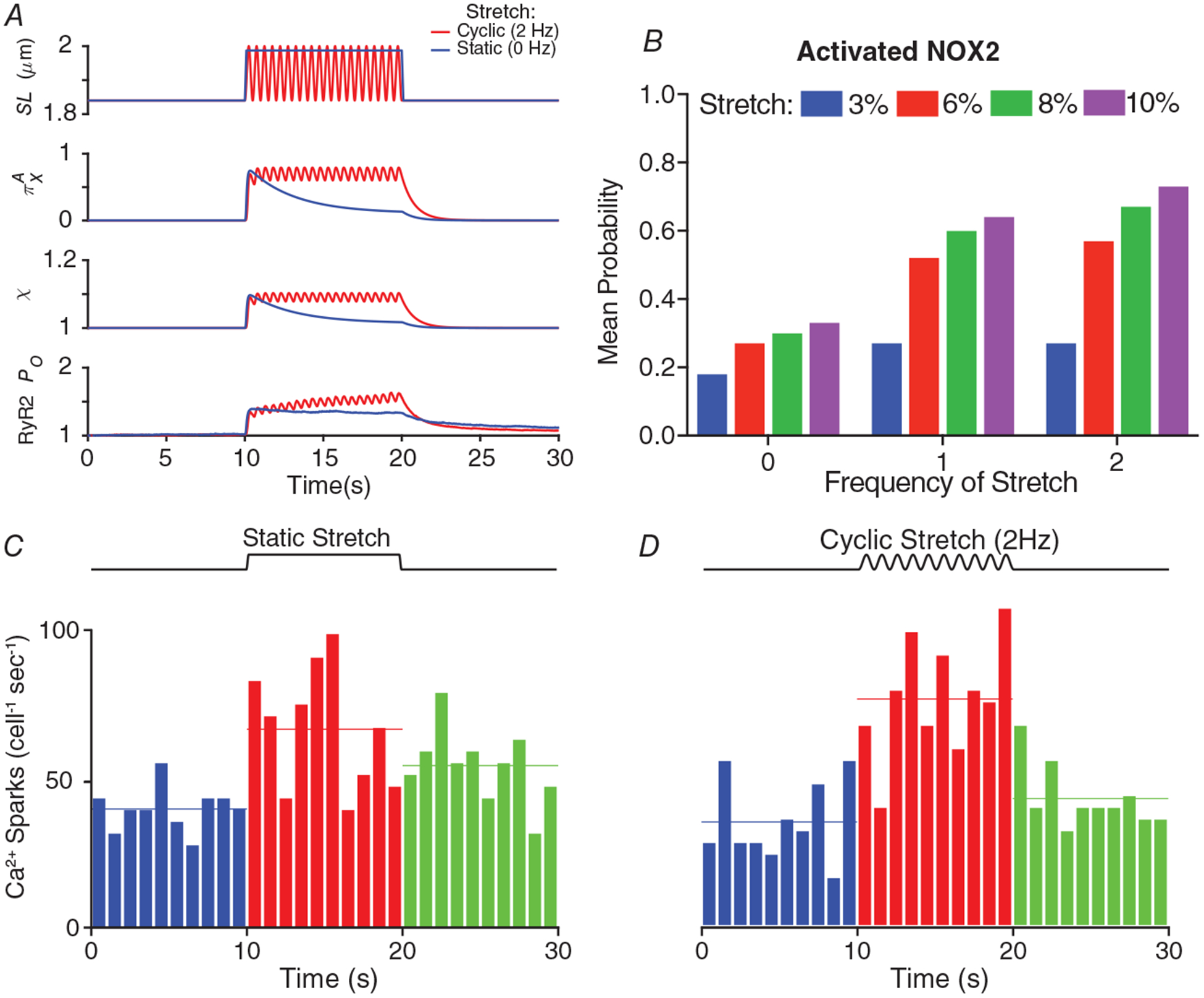
Simulating stretch-dependent changes in Ca^2+^ signalling in heart *A*, sarcomere length during a static (0 Hz, blue) and cyclical (2 Hz, red) stretch protocol, the instantaneous probability of NOX2 being in the activated state (*π*_X_^A^, see Eqn (2)), X-ROS factor (*χ*), and the instantaneous change in single RyR2 *P*_O_. *B*, the mean probability of NOX2 being in the activated state (*π*_X_^A^) during a 10 s stretch of varying magnitudes (i.e. 0, 3, 6, 8 and 10%) and frequencies (i.e. 0, 1 and 2 Hz). Note that a 0 Hz stretch indicates a static, ‘stretch-and-hold’ protocol. *C* and *D*, number of Ca^2+^ sparks (cell^−1^ s^−1^) during a static (*C*) and a cyclical (*D*) stretch protocol.

We adopted a modified manganese (Mn^2+^) quench technique to assess the magnitude of Ca^2+^ influx in Fura-2-loaded VCMs both at rest and with stretch imposed by our single myocyte stretch device (see Methods). Before stretch, each cardiomyocyte was adjusted to a consistent SL of (1.84 *μ*m). Immediately upon application of Mn^2+^ containing perfusate, cation influx was observed via quenching of Fura-2 (see Slope 1 of Fig. 3A). Upon initiation of a cyclical stretch protocol (2 Hz sinusoidal with amplitude of 10% of SL for 2 min), there was a significant increase in Mn^2+^ quench rate (see SL plot in Fig. 5A), as we previously described in skeletal muscle (Kerr *et al.* 2015). These results suggest that in the heart, physiological stretch elicits an ~2-fold increase of Ca^2+^ entry over basal conditions. Note that while basal Ca^2+^ entry is relatively small (~1 *μ*M s^−1^, see Trafford *et al.* 1997), this 2-fold change in influx will critically alter steady-state Ca^2+^ homeostasis. Finally, given that the majority of the stretch-dependent cation influx was attenuated by scavenging ROS with NAC, we conclude that stretch-dependent ROS is a potent regulator of SAC activity during stretch. This ability of ROS to regulate *I*_saca_ is reflected in Eqn (6).

### Stretch-dependent changes in Ca^2+^ sparks

We previously established that Ca^2+^ spark frequency was exquisitely sensitive to stretch via MTs and X-ROS; however, the impact on spark properties was not evaluated. Here we used a single static stretch and confocal linescan recording to profile Ca^2+^ sparks in both WT (Fig. 4A) and MDX (Fig. 4B) VCMs. Upon stretch (red arrow) we observed a rapid increase in Ca^2+^ spark frequency consistent with our previous work in MDX mice (see Prosser *et al.* 2011). Expanding upon our other work (Prosser *et al.* 2011, 2013b; Kerr *et al.* 2015), we now show that while the amplitude (Δ*F*/*F*_O_) and spatial extent (full width, half-maximum; FWHM) of Ca^2+^ sparks are insensitive to stretch, the temporal extent (full duration, half maximum; FDHM) is significantly increased (see Fig. 4C). Taken together, these results provide critical details regarding the overall amount of SR Ca^2+^ release during stretch and aided in constraining the computational model.

### Modelling stretch-dependent RyR2 activation in heart

Our approach to modelling stretch-dependent changes in Ca^2+^ signalling in heart began with our representation of NOX2 activation. Our imaging of stretch-dependent ROS (via fluorescence of H_2_-dichlorofluorescein) demonstrated that the rate of X-ROS generation is maximal immediately upon stretch and rapidly decays with either the immediate return to resting length or if the stretch is maintained (Prosser *et al.* 2011). In contrast, a cyclic stretch protocol sustains the generation of X-ROS (Prosser *et al.* 2013b). To approximate this behaviour in our model we utilized a Markov chain representation of NOX2 (see Eqn (2)). Here the mechanical stress of stretch imparts a strain in the MTs (*β*_MT_) that activates NOX2 to initiate a burst of ROS. If the stretch is sustained (Fig. 5A, SL, blue), NOX2 transitions into a refractory state (state R in Eqn (2)), which reduces the rate of NOX2 activity (i.e. ROS production) (Fig. 5B, blue). During a cyclical stretch perturbation (Fig. 5A, SL, red), releasing strain on the MT network allows NOX2 to recover from the refractory state (R) to the deactivated state (state D in Eqn (2)) and become available for reactivation by a subsequent stretch. The model behaviour across either sustained or cyclic stretch (Fig. 5A), or with varying amplitudes of stretch (Fig. 5B), faithfully captures the experimental behaviour that we previously observed (Prosser *et al.* 2013b).

Having established the model parameters for NOX2 activity (i.e. X-ROS), this behaviour was translated into a change in RyR2 gating (via *χ*, see Eqn (4) and Fig. 5A) where both the opening and closing rates of RyR2 (*k*_12_ and *k*_21_ in Eqn (5), respectively) are modified (see Eqn (5)) in a manner consistent with observed changes in RyR2 gating following oxidation of RyR2 by H_2_O_2_ (Boraso & Williams, 1994). This redox-dependent RyR2 gating results in a slight increase RyR2 *P*_O_ during stretch (see Fig. 5A), which in turn drives a change in Ca^2+^ spark behaviour with sustained (Fig. 5C) or cyclic stretch (Fig. 5D). While these results are consistent with our published experimental observations (Prosser *et al.* 2013b), a closer examination revealed two notable findings.

First, our analysis of both model- and experiment-derived sparks showed that the model also identified FDHM as a spark parameter sensitive to stretch (Figs 4C and 5B). Second, while our brief cyclic stretch (10 s, Fig. 5D) yielded a sustained Ca^2+^ spark activity as in our previous work (Prosser *et al.*, 2013b), the rapid cessation of Ca^2+^ spark activity upon return to rest did not align with the latency of spark cessation seen in this previous experimental work. To investigate this discrepancy, we extended our model simulation to 60 s of cyclic stretch and observed a 2-fold increase in spark frequency for at least 10 s into the post-stretch period (not shown).

### Modelling captures deleterious Ca^2+^ release in Duchenne muscular dystrophy

A major pathological sequelae of Duchenne muscular dystrophy (DMD) is cardiomyopathy and arrhythmia. In the *mdx* model of murine DMD, we linked the disease-dependent increase in MT density, the abundance of glu-tubulin and increased NOX2 expression to the deleterious increase in Ca^2+^ sparks and arrhythmogenic Ca^2+^ waves. As an empirical test of our model we sought to account for this behaviour by altering model parameters *a priori* based on our published and new experimental results.

Our work established MTs as central to the activation of NOX2 by MCT and showed that the abundance of glu-tubulin is critical to this effect. Recent elegant work in cardiomyocytes demonstrated that the level of glu-tubulin determines how MTs buckle under and bear the mechanical stress which transmits mechanical strain to regulate MCT (Robison *et al.* 2016). Given that we identified a ~50% increase in MT density in MDX (immunohistochemistry and western blot, see Fig. 2) with a proportionate increase in glu-tubulin, and a ~150% increase in NOX2 content, we implemented a scale factor for changes in MT and NOX2 density in MDX *vs*. WT (see Table 2, *ρ*_MT_ = 1.5 and *ρ*_NOX2_ = 2.5, respectively) in our model as conservative estimates for their influence on X-ROS in MDX. We also included alterations in SERCA and NCX activity associated with MDX animals (see Table 2; Fauconnier *et al.* 2010; Wang *et al.* 2015).

**Figure 6.**
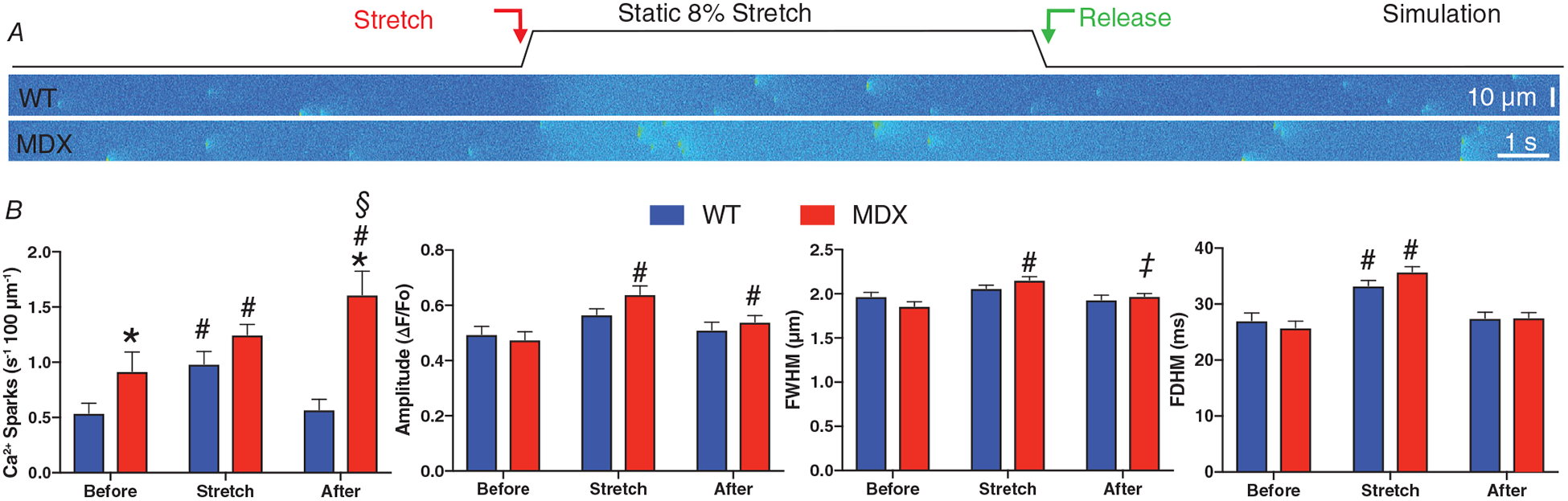
Modelling captures stretch-dependent Ca^2+^ spark behaviour in healthy and dystrophic heart *A*, representative simulations of transverse confocal linescans for WT and MDX VCMs with an 8% stretch imposed at the red arrow. *B*, Ca^2+^ spark frequency, amplitude, FDHM and FWHM before, during and after a static, 8% stretch. Note, simulated transverse linescan frequencies have been adjusted (i.e. divided by 3) to enable direct comparison to experimental longitudinal linescan measures based on an average 600 nm and 1.8 *μ*m inter-CRU distance, respectively. *C*, simulations of transverse confocal linescans for WT and MDX VCMs exposed to a cyclic, 2 Hz, 10 % stretch. Conditions as follows, WT (WT); MDX (MDX); MDX with 2-fold increase in X-ROS (MDX+); and MDX modified with ‘normalized’ (i.e, equivalent to WT levels) X-ROS components (MDX−). Statistics: MDX compared to WT (*P < 0.05), Stretch compared to Before (^#^*P* < 0.05), After compared to Before (^§^*P* < 0.05), and After compared to Stretch (^‡^*P* < 0.05).

We show the influence of static and cyclic stretch on Ca^2+^ spark activity in simulated transverse, confocal linescans from WT VCMs and see this behaviour dramatically increased in MDX (Fig. 6). In fact, cyclic stretch generates Ca^2+^ release activity that transitions from stable Ca^2+^ sparks to elevated levels of pro-arrhythmic Ca^2+^ release. This behaviour is reflected in the RyR2 integrated flux that underscores this behaviour, a behaviour driven by the model elements that impose a MT-dependent X-ROS signal that drives an increase in SAC dependent Ca^2+^ influx as well as an increase in RyR2 activation from CICR as well as luminal-dependent mechanisms (see Walker *et al.* 2014; Wescott *et al.* 2016).

### Targeting the MT network effectively suppresses Ca^2+^-dependent arrhythmias

**Figure 7.**
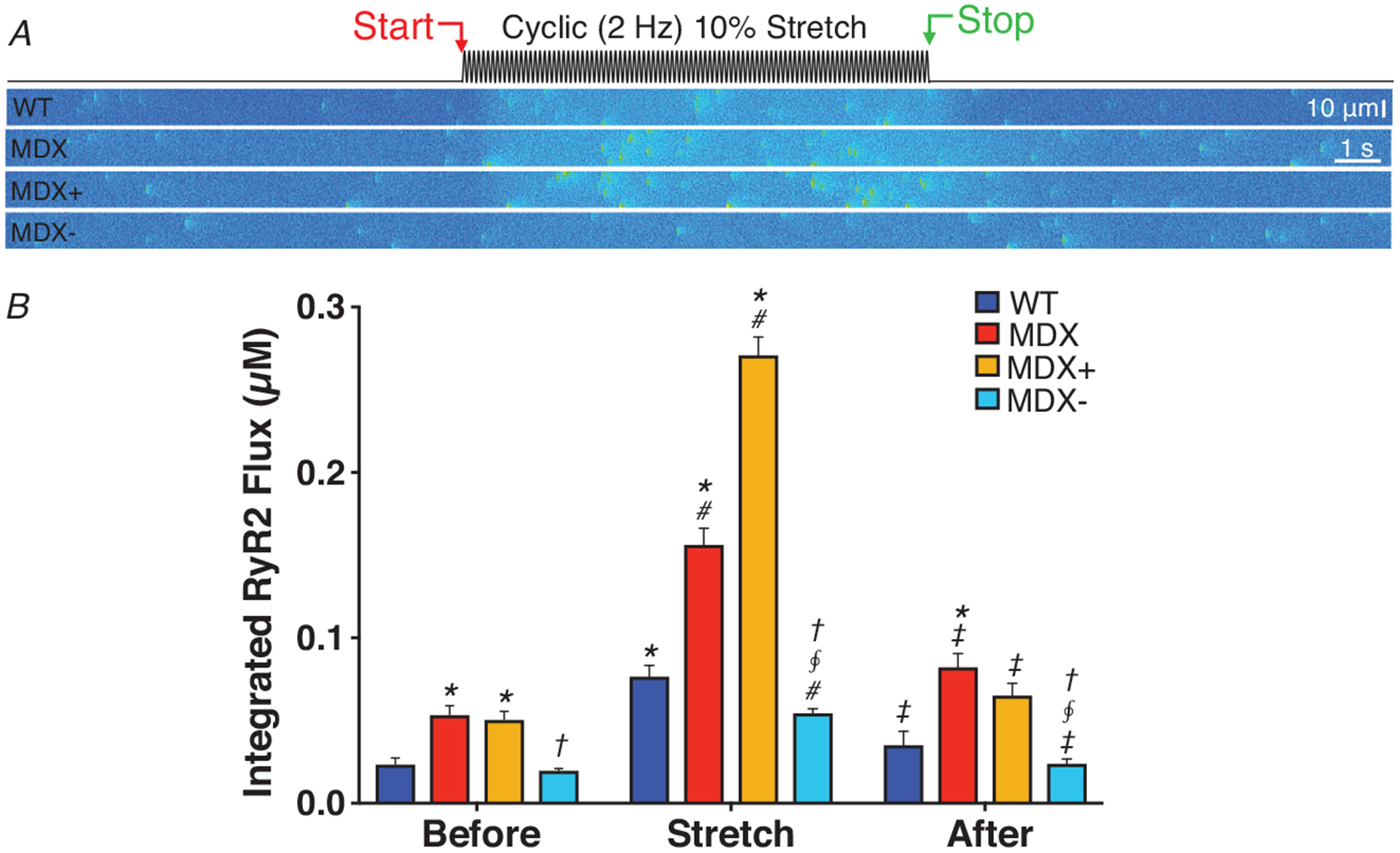
MT densification drives SR Ca^2+^ leak towards arrhythmogenic levels during cyclic stretch in heart *A*, simulations of transverse confocal linescans for WT and MDX VCMs exposed to a cyclic, 2 Hz, 10% stretch. Conditions as follows, WT (WT); MDX (MDX); MDX with 2-fold increase in X-ROS (MDX+); and MDX modified with ‘normalized’ (i.e, equivalent to WT levels) X-ROS components (MDX−). *B*, quantification of RyR release flux. Statistics: Test group (i.e. MDX, MDX+, MDX−) compared to WT (*P < 0.05) within each condition (i.e. Before, Stretch, After), MDX− compared to MDX+ within each condition (^§^*P* < 0.05), MDX− compared to MDX within each condition (^†^*P* < 0.05), Stretch compared to Before (^#^*P* < 0.05), and After compared to Stretch (^‡^*P* < 0.05).

Our evidence that targeting either MT density or its level of glu-tubulin was solely sufficient to abrogate the deleterious excess in stretch-activated X-ROS and Ca^2+^ spark activity *in vitro*, and workload elicited Ca^2+^-dependent arrhythmia *in vivo*, was firm evidence that MTs were central to disease pathology in DMD. Consistent with these published reports, our model simulations show that the normalization of MT-dependent MCT through X-ROS effectively suppressed the dysregulated excess of stretch-dependent Ca^2+^ spark activity and arrhythmogenic Ca^2+^ release in the MDX (see Figs 6C and 7).

## Discussion

We have presented a new computational model of X-ROS that tightly integrates the MT-dependent activation of NOX2 with stretch-dependent changes in Ca^2+^ signalling in heart. This model was directly informed by quantitative experimental measures and accurately captured our current and prior experimental findings that diastolic stretch elicits NOX2-dependent ROS signals which regulate Ca^2+^ sparks and cardiac ECC. Given the diverse roles played by MTs in cell physiology, our ability to account for our experimental observations with a computational model constrained by the biophysical aspects of MCT and independent of the effects of MTs on other pathways adds critical support to our hypothesis that MTs act primarily via MCT and NOX2 to regulate Ca^2+^ signalling in heart.

Notably, only a modest number of model parameters and disease-relevant scaling factors were sufficient to account for the influence of MCT through X-ROS in the healthy and diseased heart. While the ability of our computational models to faithfully account for this MCT regulation of cardiac ECC while utilizing a small number of factors is exciting, the underlying regulatory complexity within each factor prompts several questions based on this and other findings. For example, in the model, MT strain (i.e. *β*_MT_) is responsible for translating the mechanical energy of stretch to activate NOX2. However, within the cell, MT strain is determined by glu-tubulin-enriched MTs in concert with intermediate filaments (IF) and other cytolinker proteins, which also exhibit increased expression in disease. Furthermore, evidence suggests that MT and other cytoskeletal changes regulate the mechanical properties (i.e. stiffness) of the cytoskeleton to regulate MCT (Kerr *et al.* 2015; Robison *et al.* 2016). In this regard, future experimental and computational efforts directed towards understanding how cytoskeletal elements act together or separately to regulate X-ROS and how measured changes in cytoskeletal mechanics relate to the magnitude of X-ROS will greatly advance our understanding.

Our published work (Kerr *et al.* 2015) combined with new results from experiments and computational modelling provides clear evidence that MTs are essential for the activation of NOX2 via MCT. While our implementation of a three-state Markov chain faithfully captures how MT strain drives the complex behaviour of the experimentally observed X-ROS signals, whether MTs activate NOX2 by a direct mechanical means or via a MCT elicited effect (see Allen *et al.* 2016) remains unanswered.

Our experiments, and computational model, also revealed MCT through X-ROS as a regulator of mechano-sensitive sarcolemmal Ca^2+^ influx in the heathy heart and showed increased MCT through X-ROS underscores its excess in disease (Kerr *et al.* 2015). These findings add to the evidence implicating NOX2-ROS for the dysregulated sarcolemmal influx in the dystrophic heart (Gonzalez *et al.* 2014). Additionally, while MT-dependent MCT via X-ROS appears to be the dominant signal elicited by stretch, evidence from others shows that MCT-dependent RNS signals (i.e. NO) contribute to RyR2 (Petroff *et al.* 2001; Jung *et al.* 2008; Shkryl *et al.* 2009; Niggli *et al.* 2013; Jian *et al.* 2014) and TRP channel (Koitabashi *et al.* 2010; Seo *et al.* 2014a,b) regulation in heart. Whether MCT-dependent X-ROS and RNS interact independently or synergistically to RyR2 or TRPs is another unanswered question. One possible interaction is sustained Ca^2+^ spark activity observed after the cyclic stretch (Prosser *et al.* 2013b). While our simulation shows the accompanying elevation in [Ca^2+^]_i_ (by ~20 nM) and SR Ca^2+^ (by ~50 *μ*M) was a parsimonious explanation for the sustained level of spark activity seen here, we remain intrigued that this level of Ca^2+^ could also activate nNOS-dependent NO signals (Jian *et al.* 2014) that could further sustain this spark activity in this post-stretch period.

In summary, we presented a new computational model integrating MT-dependent MCT and cardiomyocyte ECC through X-ROS. In conjunction with advanced *in vitro* approaches as shown here (see Methods), this model is a powerful platform that lays the foundation for future investigations into the influence of MCT on cardiac ECC under both physiological and pathological conditions.

## Figures and Tables

**Table 1.** Select model parameters

Parameter	Description	Value (WT)	Reference/constraint
*g*_saca_	Whole conductance for stretch-activated Ca^2+^ influx pathway	1500 pS	Fig. 3
*β*_m_	MT strain scalar	10	N/A
*β*_0_	Minimal MT stain factor	0.01	N/A
*β*_1_	MT strain normalizing factor	0.1	N/A
*β*_*η*_	Cooperativity factor for MT strain	2	N/A
*ρ*_MT_	Unitless MT density	1	N/A
*ρ*_NOX2_	Unitless NOX2 density	1	N/A
*ρ*_WT_^o^	Basal oxidization factor for WT	0	Figs 3 and 4
*ρ*_MDX_^o^	Basal oxidization factor for MDX	0.08	Figs 3 and 4
*k*_da_	NOX2 rate constant	0.2 s^−1^	Prosser et al. (2011, 2013b)
*k*_ar_	NOX2 rate constant	0.25 s^−1^	Prosser *et al.* (2011, 2013b)
*k*_rd_	NOX2 rate constant	1 s^−1^	Prosser *et al.* (2011, 2013b)
*k*_ad_	NOX2 rate constant	1 s^−1^	Prosser *et al.* (2011, 2013b)
*k*_dr_	NOX2 rate constant	1 s^−1^	Prosser *et al.* (2011, 2013b)
*k*_ra_	NOX2 rate constant	0.05 s^−1^	Prosser *et al.* (2011, 2013b)
*k*_12_	RyR2 opening rate	0.2 *μ*M^−*η*^ s^−1^	Wescott *et al.* (2016)
*k*_21_	RyR2 closing rate	425 s^−1^	Wescott *et al.* (2016)
*A*_p_	SERCA density	50 *μ*M	Wescott *et al.* (2016)
*I*_ncx_^1^	Maximal NCX current	500 pA	Wescott *et al.* (2016)

See Wescott *et al.* (2016) for additional parameters.

**Table 2.** Model parameters altered in MDX

Parameter	Fold change in MDX	Reference/constraint
*ρ*_MT_	1.5	Fig. 2
*ρ*_NOX2_	2.5	Fig. 2
*A*_p_	1.5	Fauconnier *et al.* (2010), Wang et al. (2015)
*v*_ncx_	1.2	Fauconnier *et al.* (2010), Wang *et al.* (2015)

## Key points

- Our group previously discovered and characterized the microtubule mechanotransduction pathway linking diastolic stretch to NADPH oxidase 2-derived reactive oxygen species signals that regulate calcium sparks and calcium influx pathways.
- Here we used focused experimental tests to constrain and expand our existing computational models of calcium signalling in heart.
- Mechanistic and quantitative modelling revealed new insights in disease including: changes in microtubule network density and properties, elevated NOX2 expression, altered calcium release dynamics, how NADPH oxidase 2 is activated by and responds to stretch, and finally the degree to which normalizing mechano-activated reactive oxygen species signals can prevent calcium-dependent arrhythmias.
